# Digital determinants of health: Editorial

**DOI:** 10.1371/journal.pdig.0000373

**Published:** 2023-11-28

**Authors:** Hamish S. Fraser, Alvin Marcelo, Mahima Kalla, Khumbo Kalua, Leo A. Celi, Jennifer Ziegler

**Affiliations:** 1 Brown Center for Biomedical Informatics, Brown University, Providence, Rhode Island, United States of America; 2 Medical Informatics Unit, University of the Philippines, Manila, The Philippines; 3 Centre for Digital Transformation of Health, Faculty of Medicine, Dentistry and Health Sciences, University of Melbourne, Melbourne, Australia; 4 Blantyre Institute for Community Outreach and the College of Medicine, University of Malawi, Blantyre, Malawi; 5 Laboratory for Computational Physiology, Massachusetts Institute of Technology, Cambridge, Massachusetts, United States of America; 6 Division of Pulmonary, Critical Care and Sleep Medicine, Beth Israel Deaconess Medical Center, Boston, Massachusetts, United States of America; 7 Department of Biostatistics, Harvard T.H. Chan School of Public Health, Boston, Massachusetts, United States of America; 8 Department of Internal Medicine, University of Manitoba, Winnipeg, Manitoba, Canada; University of British Columbia, CANADA

Digital health systems have grown very rapidly in the last decade in high-income regions, including North America and Europe, and also in many low- and middle-income regions [1]. While core health information systems like electronic health records and radiology information systems are central to this progress, equally important are mobile health tools, telehealth systems, and certain health uses of social media. The benefits shown in healthcare delivery and public health from the use of these systems are predicated both on access to this diverse set of tools (based on access to technology, power, networking, and training) and the ability of healthcare workers and patients to use them effectively (digital literacy). In this edition of the journal, the focus is on a new concept: Digital Determinants of Health (DDoH), described below. Clearly, there are inequalities of access to healthcare and health technologies around the world and many different ways of delivering care. The concern here is with inequities in access to care driven by poverty, mismanagement, and systems that continue to be designed without a core focus on the right of all patients from all groups and abilities and in all locations to good quality health and healthcare. Health equity is “the absence of health inequities, differences in health that are unnecessary, avoidable, unfair, and unjust” [2]. Using a range of methods, the authors have explored the importance of different aspects of this emerging area of research, including surveying the literature in 3 scoping reviews, and developed new frameworks for describing and analyzing the importance of the different factors and potential biases. The comprehensiveness of this work has been aided by a policy on inclusion of authors, reviewers, and editors from different communities and countries at *PLOS Digital Health*.

DDoH is a recent concept that recognizes the increasingly important role digital health plays in access to high-quality healthcare worldwide and the factors that prevent many people from fully benefiting from these tools. Chidambaram and colleagues introduce the concept of DDoH in “An introduction to digital determinants of health” [3]. These ideas build off the existing concept of Social Determinants of Health defined as “conditions in the environments in which people are born, live, learn, work, play, worship, and age that affect a wide range of health, functioning, and quality-of-life outcomes and risks”[2]. This, in turn, is based on Dhalgren and Whitehead’s Rainbow Model [4]. They describe the derivation of DDoH, including the importance of the design, implementation, and use of technology in health. This includes emphasis on the problematic relationship between lower socioeconomic status, lower access to digital tools, and lower digital health literacy, which, in combination, can greatly reduce the benefits of digital health systems. Telehealth services are an important example in which user interface design can create challenges for older patients or those with disabilities including hearing or vision impairment, a group that also tend to have lower access to high-speed broadband. Finally, they identify the effects of atypical patients, data poverty, and asymmetry on the development and use of artificial intelligence (AI) and machine learning (ML) models. This includes underrepresentation or bias in data from underserved communities.

“Digital literacy is critically important, along with digital tools and infrastructure to ensure adoption, scalability, and impact of digital solutions. Poor digital literacy is a key factor in the DDoH.” Arias López and colleagues carried out a scoping review of “Digital literacy as a new determinant of health” [5]. The definition of Digital Health Literacy they use is “the ability to find and use health information with the goal of addressing or solving a health problem with technology.” This includes the more general concept of health literacy—understanding health information and its importance for the individual or their family, which is also lower in many disadvantaged communities. They report that a higher digital health literacy had a positive impact on patient self-management, participation in medical decision-making, psychological state, and quality of life. They describe growing interest in measurement of digital health literacy, but a need for better tools and interventions to identify and intervene to support underserved communities.

Telehealth illustrates both the benefits of rapid adoption of a valuable digital health service and the real harm that can be experienced by patients who cannot access it. Phuong and colleagues examined the access to effective telehealth services in the United States in the article “Telehealth and digital health innovations: A mixed landscape of access” [6]. The COVID pandemic drove great growth in access and use of telehealth services, breaking through many legal, policy, and financing barriers particularly in the US. However, access to these services remains uneven, with underserved groups receiving less access due to many factors, including poor broadband and technology access, reduced digital literacy, and even unstable access to electrical power. To improve effective and equitable access to telehealth services, they highlight the need for more flexible, accessible, useable, and robust telehealth systems.

Digital health systems often perform differently in different patient groups, and these biases can lead to false confidence in the results and therefore poorer patient care. Charpignon and colleagues analyzed some of the underlying processes that may worsen disparities in the benefits seen from digital health in “Going beyond the means: Exploring the role of bias from digital determinants of health in technologies” [7]. Their focus is on the technologies and medical formulae (excluding AI and ML) and finding evidence of deficiencies in performance of these technologies across groups. Mechanisms of deficiencies and biases were grouped into physical and biological (e.g., the effects of skin color on pulse oximetry), interactions of human factors and cultural practices (e.g., in the use of electroencephalography), and interpretation bias (e.g., the effects of patient characteristics on the interpretation of pulmonary function tests). Many of the technologies examined showed lower accuracy or validity in specific patient groups especially those outside the original scope of development. They recommend approaches to increasing the diversity of developers of technology and patient groups, and for clinicians to be alert to the need to question results and cross-correlate with other methods.

A key factor in determining the effectiveness of digital health and especially ML models is data completeness and accuracy, including how faithfully it represents all communities and individuals. Paik and colleagues carried out a scoping review entitled “Health data poverty amplifies existing health disparities” [8]. Their focus included the impact of health data disparities on the development and validation of AI and ML algorithms, and the risk of bias that can occur when those algorithms are deployed for patient care in disadvantaged communities. More than two-thirds of studies identified addressed health disparities, followed by studies of AI/ML bias, and biases in the input data for development of the algorithms. They identified only 3 studies from low- and middle-income countries indicting a need for more research globally, particularly in the most vulnerable communities. They stress the importance of collecting comprehensive unbiased data, better understanding of limitations of ML algorithms, and need for more evaluation and regulation before and after deployment of AI and ML algorithms.

ML algorithms have great promise for improving healthcare, but they are prone to biases that can affect their performance and safety. Nazer and colleagues address these concerns in an article entitled “Bias in Artificial Intelligence Algorithms and Recommendations for Mitigation” [9]. They specifically examined the interaction of social determinants of health, algorithm behavior, and health outcomes, and explored the ways that biases may affect each step in algorithm development. They identified strategies to address biases from the stages of (1) framing the problem; (2) data sources; (3) data preprocessing; (4) model development; (5) model validation; and (6) model implementation, and developed a checklist to support reducing bias during each of these stages. They identity a lack of understanding of sources of bias particularly in low- and middle-income countries, and they recommend a diverse team to oversee AI/ML work there.

“Strategies and Solutions to Address Digital Determinants of Health (DDoH) across Underinvested Communities” is the title of the last article, a scoping review carried out by Homes Fee and colleagues [10]. They highlight the tension between the goal of improving services through digital health technologies and the risk that these solutions will increase disparities in care. The review focused on solutions shown to reduce such disparities, which were categorized into (1) policy; (2) design and development; (3) implementation and adoption; and (4) evaluation and ongoing monitoring. They identified gaps in the current literature, emphasizing the need for monitoring and evaluation of the effectiveness of these solutions to address DDoH. Common strategies and solutions to address negative impacts of DDoH were identified, and a framework proposed for DDoH assessment to improve design and deployment of digital health.

Overall, these studies provide a wide-ranging view of this increasingly important issue and some of the strategies that may be used to address it. They also illustrate the importance of further research both for understanding the scale and nature of the problem and to outline possible strategies and tools to address it. DDoH are important in identifying and understanding key challenges in working with underserved populations in high-income countries whether they are rural populations, particular ethnic groups, people with disabilities that impact IT use, or patients with limited health literacy. Applying frameworks based on DDoH also helps identify serious problems in low- and middle-income countries and remote areas worldwide. This includes data poverty that impacts on access to care, quality of care, clinical research, and the representativeness, effectiveness, and safety of ML models built with this data. Without this approach, deployment of digital health systems risks replacing one set inequalities of care with another. Researchers, clinicians, funders, ministries of health, and journals should work to ensure concepts of DDoH are built into future projects and publications.
